# Systematic analysis of the global, regional, and national burden of subarachnoid hemorrhage from 1990 to 2021

**DOI:** 10.1371/journal.pone.0323453

**Published:** 2025-05-29

**Authors:** Wanyue Li, Huafei Yang, Furong Rui, Xinyi Ruan, Jun Xiong, Lin Chen

**Affiliations:** 1 School of Public Health and Nursing, Hangzhou Normal University, Hangzhou, Zhejiang, China; 2 School of Public Management, Hangzhou Normal University, Hangzhou, Zhejiang, China; 3 Engineering Research Center of Mobile Health Management System, Ministry of Education, Hangzhou, Zhejiang, China; Indiana University School of Medicine, UNITED STATES OF AMERICA

## Abstract

Subarachnoid hemorrhage (SAH) is characterized by high rates of morbidity, mortality, and disability, which imposes a heavy disease burden on society and families. Incidence, mortality, disability-adjusted life years (DALYs) and their corresponding age-standardized rates (ASRs) and estimated annual percentage changes (EAPCs) of subarachnoid hemorrhage were analyzed by using GBD 2021 data in our study. Data were further stratified by age, sex, and region. Globally, in 2021, there were 697486 (95% UI, 614334795785) new cases of subarachnoid hemorrhage, 352,810 (309015401474) deaths, and 10.64 million (9.39 to 12.12) DALYs. The burden of subarachnoid hemorrhage showed substantial decline from 1990 to 2021. However, the decline in the age-standardized incidence rate was significantly smaller than the declines in the age-standardized mortality rate and age-standardized DALY rate, with the most pronounced downward trend in East Asia but an increase in sub-Saharan Africa, indicating that our prevention strategies require further refinement.

## Introduction

Subarachnoid hemorrhage is defined as a non-traumatic stroke caused by bleeding into the subarachnoid space, typically resulting in permanent damage to the central nervous system [1]. The Global Burden of Diseases, Injuries, and Risk Factors study shows that stroke will remain the third leading cause of death [2]and the fourth leading cause of disability [3]worldwide in 2021 in the context of the COVID-19 pandemic. In 2021, the global age-standardized incidence rate of subarachnoid hemorrhage was 8.33/100,000 (95% UI, 7.34 to 9.48), the age-standardized mortality rate was 4.19/100,000 (3.66 to 4.76), and the age-standardized DALY rate was 125.20/100,000 (110.54 to 142.61). Although subarachnoid hemorrhage is less prevalent than ischemic stroke and cerebral hemorrhage, it has high morbidity and mortality rates. Additionally, survivors may experience cognitive deficits and mental health symptoms, such as depression and anxiety, which can be long-lasting [4–6], so its potential impact on the patient as an individual, their families and communities should not be overlooked.

Past studies have mostly focused on stroke overall in global, regional, and national releases, and ischemic stroke as the main type of stroke has attracted much attention, but systematic studies of the global, regional and national burden of disease for subarachnoid hemorrhage are lacking. With evolving disease patterns and demographics worldwide, as well as dynamic changes in life expectancy and mortality, epidemiologic data on subarachnoid hemorrhage needs to be updated, which includes key indicators such as incidence, mortality, disability, risk factors, and prevalence trends. This study aims to fill this gap by providing up-to-date epidemiological data that can be used to optimize the allocation of healthcare resources and improve the effectiveness of public health interventions, as well as helping to promote evidence-based healthcare practices, improve patient prognosis and quality of life, and reduce the burden of disease.

## Methods

### Data sources

The newly released GBD 2021 report provides a comprehensive dataset covering epidemiological information from 1990 to 2021 that includes, but is not limited to, the number of new cases, prevalent cases, number of deaths, disability-adjusted life years (DALYs), years of life lost (YLLs), years of life with disability (YLDs), and the corresponding age-standardized rates for different ages, genders, regions, and years; covers 288 causes of death in 25 age groups from birth to 95 years and over, and the health status of males, females, and both sexes in 204 countries and territories in 21 regions and 7 super-regions [2].The GBD database is based on a wide range of data sources, including vital registration systems, population censuses, disease-specific registries, and health service linkage data. It focuses on 371 diseases and injuries and provides association analysis of 88 risk factors with 631 risk-outcome pairs [3,7]. We performed a secondary analysis of GBD2021, and extract detailed data on the burden of subarachnoid hemorrhage (SAH) from the GBD 2021 database (https://vizhub.healthdata.org/gbd-results/), including incidence, deaths, disability-adjusted life years (DALYs), and their corresponding age-standardized rates, at the global, regional, and country levels from 1990 to 2021.The rates are further stratified by sex, age, region, and country.

### Terms and definitions

**Age-standardized rate** Rate per 100,000 population following standardization to the global age structure. The difference between age-standardized rates across geographies and over time is independent of population size and age structure.

**ASIR**: Age standardized incidence rate, a standardized incidence rate index based on age, which is used to compare the incidence rate of different age groups.

**ASMR** Age standardized mortality rate, is an indicator obtained by comparing the mortality rate of different age groups with the age structure of the standard population.

**ASDR**: Age standardized DALYs rate, which is used to standardize DALYs for comparison between different populations or time periods.

**EAPC**: Estimated annual percentage change, quantitatively presenting disease burden based on GBD longitudinal data, reflecting the overall trend of ASDR changes.

### Statistical analysis

Subarachnoid hemorrhage incidence, mortality, prevalence, and DALY estimates were expressed as absolute numbers and age-standardized rates (with a 95% UI) per 100,000 population, stratified by age, sex, and 21 GBD regions. For the purpose of describing and evaluating the burden of global, regional and national subarachnoid hemorrhage from 1990 to 2021, ASR and EAPC were adopted in our study. By using ASR, age standardized rates were obtained to facilitate the comparison of incidence rate data in different regions or periods. EAPC is an estimate of the annual percent change over a specific period of time estimated by a linear regression model to quantify the average annual rate of change over a specific period of time. The specific method is: [ln (ASR) =*α* +* β*× (calendar year) +*ε*], *α* denotes the intercept, *ε* represents the error term, and *β* reflects a linear positive or negative trend in ASR. Both the EAPC value and the lower limit of 95% UI were greater than 0, indicating an upward trend in ASR. On the contrary, both the EAPC value and upper limit were less than 0, indicating a decreasing trend in ASR. When 95% of the UI contains 0, it indicated a constant trend of ASR. Statistical analysis was conducted using R software (version 4.3.2) and the JD_GBDR-V2-04_packed analysis tool. And a two-sided *P* < 0.05 was considered statistically significant.

## Results

### Global level

In 2021, 697486.493 (95% UI: 614334.164 to 795785.259) new cases of subarachnoid hemorrhage, 352810.217 (309015.350 to 401473.543) deaths and 10641881.907 (9398962.530 to 12121262.538) Disability Adjusted Life Years (DALY) (Table 1 Table 2 Table 3)was recorded. In the same year, the global age-standardized incidence rate (ASIR) of subarachnoid hemorrhage was 8.33 (95% UI: 7.34 to 9.48) per 100,000 population (Table 1); the age-standardized mortality rate (ASMR) and the age-standardized DALY rate (ASDR) were 4.19 (95% UI: 3.66 to 4.76) and 125.20 (95% UI: 110.54 to 142.61) (Table 2 Table 3).

**Table 1:** Global incidence number and age standardized incidence rate of subarachnoid hemorrhage from 1990 to 2021 and their temporal changes.

Characteristics	1990		2021		1990-2021	
Incident cases No.*10^3^(95%UI)	ASIR (per 100,000) No.(95%UI)	Incident cases No.*10^3^(95%UI)	ASIR (per 100,000) No.(95%UI)	Percentage change	EAPC No.(95%UI)
Global	508.79(441.50-587.62)	11.69(10.22-13.51)	697.49(614.33-795.79)	8.33(7.34-9.48)	37.09%	-1.52(-1.67--1.36)
Andean Latin America	4.55(4.01-5.07)	16.31(14.45-18.4)	7.84(7.00-8.78)	12.29(11.04-13.73)	72.53%	-0.99(-1.05--0.92)
Australasia	1.69(1.51-1.90)	7.64(6.84-8.57)	2.63(2.33-2.99)	6.06(5.34-6.86)	55.56%	-0.80(-0.84--0.76)
Caribbean	3.43(3.01-3.84)	11.47(10.09-12.93)	5.33(4.78-6.04)	10.41(9.34-11.71)	55.25%	-0.43(-0.50--0.37)
Central Asia	4.95(4.31-5.54)	9.30(8.21-10.54)	7.81(6.96-8.73)	8.97(8.10-9.98)	57.86%	-0.05(-0.08--0.01)
Central Europe	12.99(11.53-14.60)	9.48(8.45-10.59)	12.71(11.52-13.98)	7.27(6.55-8.02)	-2.15%	-1.14(-1.23--1.04)
Central Latin America	13.69(12.04-15.34)	11.85(10.46-13.41)	28.47(25.35-32.27)	11.07(9.92-12.52)	107.98%	-0.28(-0.30--0.25)
Central Sub-Saharan Africa	2.48(2.12-2.92)	7.76(6.61-9.26)	6.07(5.22-7.07)	7.29(6.26-8.51)	144.47%	-0.25(-0.28--0.21)
East Asia	154.86(132.43-181.54)	17.74(15.21-20.84)	151.82(131.56-176.50)	7.89(6.94-9.03)	-1.96%	-3.60(-3.98--3.20)
Eastern Europe	25.11(21.51-29.21)	9.83(8.52-11.38)	27.74(24.53-31.83)	9.59(8.42-10.87)	10.46%	-0.27(-0.53--0.01)
Eastern Sub-Saharan Africa	9.67(8.32-11.21)	8.99(7.66-10.75)	18.58(15.96-21.45)	7.25(6.25-8.62)	92.18%	-0.90(-0.97--0.82)
High-income Asia Pacific	34.84(30.00-41.21)	17.27(14.98-20.32)	46.62(41.13-53.65)	14.09(12.30-16.40)	33.80%	-0.73(-0.80--0.66)
High-income North America	22.73(19.66-26.71)	7.16(6.19-8.35)	33.65(29.72-38.72)	6.37(5.56-7.31)	48.04%	-0.55(-0.72--0.37)
North Africa and Middle East	20.48(17.70-23.13)	8.73(7.67-9.91)	32.39(28.11-36.31)	5.97(5.27-6.66)	58.17%	-1.54(-1.63--1.43)
Oceania	0.61(0.53-0.69)	14.87(13.23-16.94)	1.27(1.12-1.42)	12.52(11.25-13.97)	108.58%	-0.70(-0.74--0.65)
South Asia	79.74(68.84-92.81)	10.91(9.42-12.90)	140.53(120.48-162.71)	8.44(7.33-9.75)	76.24%	-1.12(-1.23--1.01)
Southeast Asia	42.84(37.09-48.90)	13.63(11.96-15.65)	74.81(65.47-85.58)	10.89(9.66-12.36)	74.65%	-0.89(-0.96--0.82)
Southern Latin America	7.57(6.75-8.61)	16.15(14.42-18.35)	8.42(7.55-9.48)	10.65(9.51-11.93)	11.17%	-1.52(-1.64--1.39)
Southern Sub-Saharan Africa	2.10(1.79-2.42)	5.82(5.00-6.84)	3.59(3.11-4.14)	5.26(4.59-6.10)	71.25%	-0.41(-0.52--0.30)
Tropical Latin America	16.72(14.08-19.46)	13.93(11.82-16.44)	26.00(22.46-30.29)	10.21(8.87-11.79)	55.52%	-1.36(-1.51--1.21)
Western Europe	39.38(34.66-45.31)	8.12(7.10-9.23)	43.95(39.40-49.55)	6.31(5.57-7.13)	11.60%	-0.90(-0.95--0.85)
Western Sub-Saharan Africa	8.38(7.16-9.58)	6.04(5.19-7.09)	17.26(14.76-19.80)	5.00(4.34-5.84)	106.06%	-0.81(-0.89--0.73)

**Table 2:** Global deaths and age standardized mortality rates from subarachnoid hemorrhage from 1990 to 2021 and their temporal changes.

Characteristics	1990		2021		1990-2021	
Death cases No.*10^3^(95%UI)	ASMR (/100,000) No.(95%UI)	Death cases No.*10^3^(95%UI)	ASMR (/100,000) No.(95%UI)	Percentage change	EAPC No.(95%UI)
Global	374.89(270.97-465.03)	9.54(6.81-11.91)	352.81(309.02-401.47)	4.19(3.66-4.76)	-5.89%	-3.09(-3.31--2.86)
Andean Latin America	1.98(1.67-2.47)	8.08(6.77-10.34)	3.64(2.95-4.42)	6.01(4.87-7.29)	83.99%	-1.03(-1.18--0.87)
Australasia	1.03(0.97-1.09)	4.54(4.26-4.80)	1.32(1.16-1.43)	2.48(2.22-2.68)	28.33%	-1.96(-2.07--1.85)
Caribbean	1.82(1.49-2.14)	6.47(5.37-7.76)	2.63(2.04-3.27)	5.03(3.88-6.25)	44.57%	-0.75(-0.87--0.64)
Central Asia	2.15(1.92-2.40)	4.59(4.10-5.16)	4.01(3.65-4.45)	5.20(4.73-5.76)	86.44%	0.54(0.32-0.76)
Central Europe	7.79(7.35-8.23)	5.43(5.13-5.75)	7.01(6.43-7.55)	3.39(3.11-3.65)	-9.95%	-1.65(-1.92--1.37)
Central Latin America	4.24(4.11-4.42)	4.37(4.20-4.59)	11.95(10.60-13.37)	4.76(4.22-5.31)	181.66%	0.40(0.25-0.55)
Central Sub-Saharan Africa	0.89(0.47-1.83)	3.60(1.66-7.88)	1.85(0.77-5.02)	3.16(1.24-8.95)	106.60%	-0.48(-0.56--0.40)
East Asia	191.91(92.73-251.48)	26.47(12.55-34.94)	95.18(70.28-119.16)	4.71(3.49-5.87)	-50.40%	-6.56(-7.23--5.88)
Eastern Europe	14.36(13.45-15.19)	5.55(5.18-5.88)	18.20(16.68-19.68)	5.49(5.03-5.93)	26.72%	-0.72(-1.69-0.26)
Eastern Sub-Saharan Africa	3.68(1.37-9.33)	4.49(1.58-11.95)	5.58(2.11-15.46)	2.92(1.10-8.24)	51.80%	-1.55(-1.61--1.50)
High-income Asia Pacific	17.90(16.56-19.79)	8.94(8.23-9.89)	17.74(14.89-19.53)	3.90(3.48-4.20)	-0.91%	-2.90(-3.00--2.79)
High-income North America	12.54(11.85-12.96)	3.78(3.59-3.91)	19.71(17.78-20.83)	3.15(2.87-3.32)	57.14%	-0.58(-0.73--0.42)
North Africa and Middle East	11.47(7.93-17.66)	6.24(4.05-10.33)	12.26(9.70-15.96)	2.85(2.28-3.69)	6.86%	-2.61(-2.75--2.47)
Oceania	0.39(0.28-.53)	12.34(8.71-17.38)	0.70(0.50-.96)	8.61(6.03-11.95)	79.47%	-1.21(-1.25--1.18)
South Asia	42.28(25.79-67.71)	6.77(3.93-11.54)	67.62(48.40-93.44)	4.47(3.14-6.23)	59.93%	-1.38(-1.43--1.33)
Southeast Asia	23.76(18.82-33.54)	9.29(7.28-13.69)	37.58(31.54-51.34)	6.02(5.02-8.42)	58.19%	-1.46(-1.55--1.37)
Southern Latin America	4.55(4.16-4.84)	9.89(9.04-10.54)	3.52(3.25-3.74)	4.13(3.82-4.39)	-22.56%	-2.68(-2.91--2.46)
Southern Sub-Saharan Africa	0.53(0.45-.69)	1.82(1.52-2.47)	1.22(1.01-1.57)	2.05(1.70-2.62)	131.14%	0.67(0.27-1.07)
Tropical Latin America	8.11(7.86-8.38)	7.47(7.21-7.74)	14.03(13.23-14.63)	5.45(5.12-5.68)	73.14%	-1.06(-1.27--0.85)
Western Europe	20.41(19.18-21.26)	3.81(3.59-3.96)	21.91(19.17-23.39)	2.28(2.07-2.41)	7.34%	-1.51(-1.72--1.30)
Western Sub-Saharan Africa	3.10(1.55-6.82)	3.18(1.52-7.14)	5.15(2.60-11.59)	2.22(1.12-5.09)	66.02%	-1.33(-1.44--1.22)

**Fig 1:**
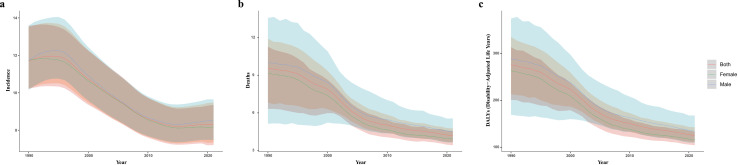
Temporal trends in global age-standardized rates (per 100,000) of subarachnoid hemorrhage from 1990 to 2021. (a) Age-standardized incidence rates; (b) age-standardized mortality rates; (c) age-standardized DALYs rates. Shading represents the 95% UI of the corresponding rates.

Both ASMR and ASDR for subarachnoid hemorrhage showed a uniform downward trend globally from 1990–2021, with ASMR and ASDR being slightly higher in males than in females, but there was no significant difference in the downward trend between the two genders (Fig 1 b and c). However, the global downward trend in ASIR for subarachnoid hemorrhage only lasted until 2015, entering a plateau from 2015 to 2021 (Fig 1a). More specifically, from 1990 to 2021, the annual percentage change in ASIR was -1.52 (95% UI: -1.67 to -1.36) (Table 1); the EAPC for ASMR was -3.09 (95% UI: -3.31 to -2.86) (Table 2); while the EAPC for ASDR was -2.88 (95% UI: -3.07 to -2.70) (Table 3).

**Table 3:** Global DALYs caused by subarachnoid hemorrhage and age standardized DALY rates and their temporal changes from 1990 to 2021.

Characteristics	1990		2021		1990-2021	
DALYs No.*10^4^(95%UI)	ASDR (/100,000) No.(95%UI)	DALYs No.*10^4^(95%UI)	ASDR (/100,000) No.(95%UI)	Percentage change	EAPC No.(95%UI)
Global	1203.13(940.98-1450.79)	275.85(213.22-335.43)	1064.19(939.90-1212.13)	125.20(110.54-142.61)	-11.55%	-2.88(-3.07--2.70)
Andean Latin America	8.64(7.37-10.37)	294.97(251.52-358.75)	12.65(10.54-15.16)	199.49(166.06-238.99)	46.37%	-1.32(-1.45--1.18)
Australasia	3.18(3.01-3.35)	142.08(134.44-149.59)	3.33(3.07-3.58)	73.27(68.07-78.65)	4.73%	-2.16(-2.24--2.08)
Caribbean	7.58(6.01-8.67)	244.64(198.19-281.75)	9.63(7.36-11.86)	191.37(146.14-234.04)	27.04%	-0.67(-0.80--0.53)
Central Asia	7.46(6.81-8.24)	144.02(131.09-159.70)	12.59(11.29-13.98)	143.22(129.05-158.53)	68.85%	-0.05(-0.22-0.13)
Central Europe	26.89(25.45-28.38)	189.14(179.14-199.28)	18.94(17.46-20.38)	106.16(97.77-114.30)	-29.59%	-2.00(-2.23--1.75)
Central Latin America	18.14(17.44-18.94)	155.66(149.23-163.02)	39.22(35.03-44.04)	151.45(135.49-170.06)	116.16%	-0.02(-0.10-0.07)
Central Sub-Saharan Africa	3.90(2.42-7.16)	116.51(64.04-228.00)	7.48(3.71-18.23)	99.08(47.49-248.36)	91.92%	-0.57(-0.63--0.51)
East Asia	537.41(285.89-688.34)	601.42(311.16-777.69)	239.70(184.07-293.41)	116.37(89.68-141.75)	-55.40%	-6.17(-6.78--5.55)
Eastern Europe	44.36(41.91-46.52)	168.44(159.14-176.74)	49.47(45.70-53.25)	162.91(150.36-175.08)	11.52%	-0.74(-1.49-0.01)
Eastern Sub-Saharan Africa	15.03(6.76-34.22)	143.75(60.68-343.61)	23.48(10.50-58.93)	96.66(42.93-245.35)	56.16%	-1.44(-1.49--1.38)
High-income Asia Pacific	57.79(53.63-63.57)	284.15(263.54-312.76)	48.52(43.41-53.18)	135.70(124.59-146.65)	-16.04%	-2.54(-2.60--2.47)
High-income North America	41.71(40.11-43.30)	132.27(127.27-137.17)	52.55(49.53-55.47)	97.82(92.56-103.14)	26.00%	-1.01(-1.11--0.92)
North Africa and Middle East	49.93(38.36-68.06)	196.72(142.41-290.27)	43.39(35.17-54.70)	82.79(67.60-104.61)	-13.10%	-2.90(-3.01--2.78)
Oceania	1.75(1.28-2.28)	391.57(288.24-525.24)	3.10(2.36-4.01)	285.62(209.42-379.65)	77.48%	-1.05(-1.07--1.02)
South Asia	164.01(111.37-240.99)	215.41(138.10-334.01)	236.28(177.21-312.70)	142.60(106.78-189.60)	44.06%	-1.35(-1.38--1.30)
Southeast Asia	88.59(73.11-114.85)	277.75(225.68-373.68)	127.94(109.43-163.70)	182.03(155.77-235.86)	44.42%	-1.39(-1.46--1.32)
Southern Latin America	15.70(14.58-16.62)	333.03(309.20-352.45)	10.82(10.10-11.48)	134.19(125.36-142.61)	-31.12%	-2.85(-3.02--2.66)
Southern Sub-Saharan Africa	2.22(1.91-2.77)	64.73(55.34-81.71)	4.70(3.93-5.89)	69.35(58.45-86.11)	111.45%	0.50(0.14-0.85)
Tropical Latin America	34.11(33.08-35.17)	283.45(274.62-292.57)	46.08(44.02-47.92)	177.53(169.49-184.75)	35.07%	-1.64(-1.82--1.47)
Western Europe	61.63(58.92-64.16)	126.14(120.93-131.23)	51.13(47.35-54.44)	67.75(63.56-71.94)	-17.05%	-1.97(-2.12--1.81)
Western Sub-Saharan Africa	13.09(7.59-25.99)	107.46(57.78-225.15)	23.21(13.72-46.48)	77.81(44.39-161.92)	77.23%	-1.17(-1.27--1.07)

**Fig 2:**
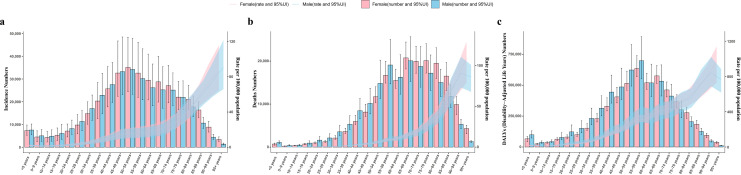
Gender and age structure analysis of the global burden of disease for subarachnoid hemorrhage in 2021. (a) incidence rates; (b) mortality rates; (c) DALYs rates. Error lines indicate 95% uncertainty intervals (95% UI) for the number of cases. Shading indicates the 95% UI of the corresponding rates.

In terms of sex-age, although mortality and DALY rates for subarachnoid hemorrhage in males peaked at 90–94 years of age and then declined, overall the incidence, mortality, and DALY rates for subarachnoid hemorrhage in both males and females demonstrated an increase in age and were higher in males than in females in most age groups (Fig 2).The incidence of subarachnoid hemorrhage was higher in females than in males in the under 5 years and 50–69 years age groups, and showed to be higher in males in the other age groups (Fig 2a). In terms of mortality, females had a higher than males only after the age of 95 years (Fig 2b), whereas the DALY rate was higher in females aged 75 years and older(Fig 2c).

### Regional level

Comparing the burden of disease for subarachnoid hemorrhage across 21 regions globally, the high-income Asia-Pacific region had the highest age-standardized incidence rate (ASIR) for subarachnoid hemorrhage in 2021 at 14.09 per 100,000 people (95% UI: 12.30 to 16.40), followed by Oceania at 12.52 per 100,000 people (95% UI: 11.25 to 13.97) and 12.29 per 100,000 (95% UI: 11.04 to 13.73) in Andean Latin America (Table 1). Meanwhile Oceania had the highest age-standardized mortality rate (ASMR) and DALY rate (ASDR) of 8.61 per 100,000 (95% UI: 6.03 to 11.95) and 285.62 per 100,000 (95% UI: 209.42 to 379.65).Andean Latin America also ranked in the top three for both ASMR and ASDR, while the high-income Asia-Pacific region, which had the highest ASIR for subarachnoid hemorrhage in 2021, ranked the 12/21 and 10/21 levels of ASMR and ASDR (Table 2 Table 3), while regions with a high burden of disease for subarachnoid hemorrhage, such as Sub-Saharan Africa, were at relatively lower levels.

Between 1990 and 2021, all 21 regions of the world showed a decreasing trend in ASIR for subarachnoid hemorrhage with an EAPC and its upper limit of UI less than 0 (Table 1), and the most pronounced decreasing trend in East Asia, with an EAPC of -3.60 (95% UI: -3.98 to -3.20). Declining trends were also more pronounced in North Africa and the Middle East (EAPC = -1.54 (-1.63 to -1.43)) and Southern Latin America (EAPC = -1.52 (-1.64 to -1.39)) more pronounced. Age-standardized mortality rates for subarachnoid hemorrhage showed a decreasing trend between 1990 and 2021 in 18 regions globally, such as East Asia (EAPC = -6.56 (95% UI: -7.23 to -5.88)), high-income Asia-Pacific (EAPC = -2.90 (-3.00 to -2.79)), and Southern Latin America (EAPC = -2.68 (-2.91 to -2.46)). However, ASMR was elevated in Sub-Saharan Africa, Central Latin America, and Central Asia, with EAPCs of 0.67 (0.27 to 1.07), 0.54 (0.32 to 0.76), and 0.40 (0.25 to 0.55), respectively (Table 2).The age-standardized DALY rates for 1990–2021 were only shown to be elevated in Sub-Saharan Africa (EAPC of 0.50 (95% UI: 0.14 to 0.85)) declined as well in the other 20 regions (Table 3), with East Asia’s declining trend in ASDR (EAPC = -6.17 (-6.78 to -5.55)) at the top of the list of the 21 regions, consistent with the EAPCs for both ASIR and the ASMR (Table 1 Table 2 Table 3).

### Country level

**Fig 3:**
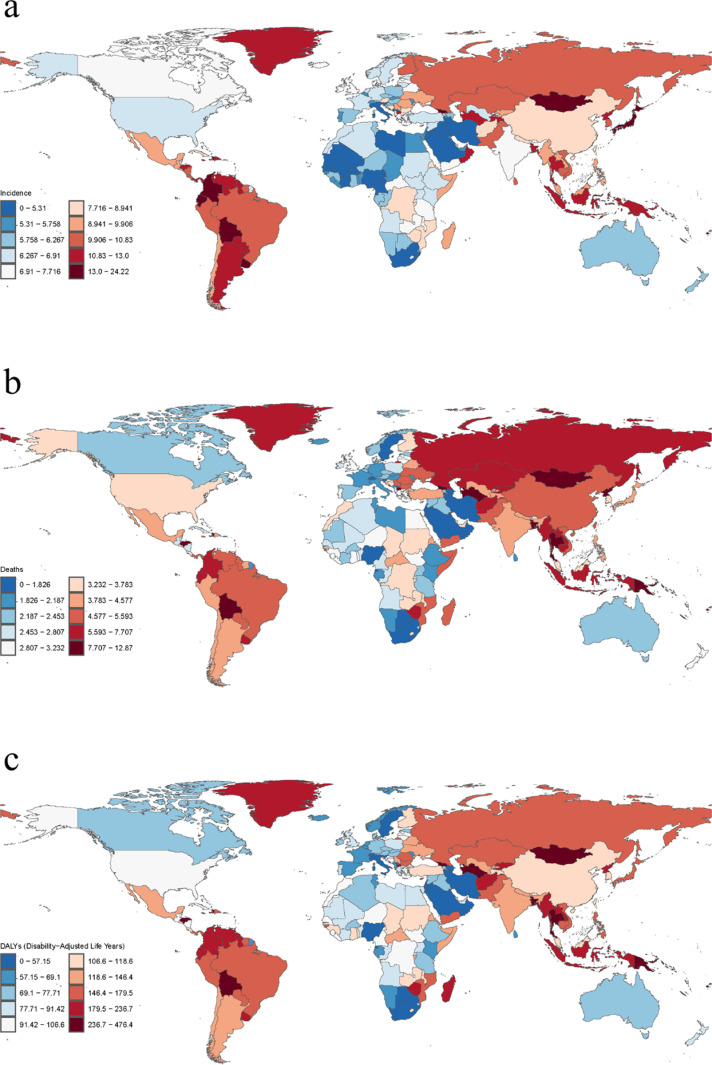
Age-standardized rates of subarachnoid hemorrhage (per 100,000 population) by country in 2021. (a) Age-standardized incidence rates; (b) age-standardized mortality rates; and (c) age-standardized rates of DALYs.

Among the 204 countries and territories, Solomon Islands had the highest age-standardized incidence rate (ASIR) of subarachnoid hemorrhage, followed by Kiribati, Marshall Islands, Federated States of Micronesia, and Vanuatu (Fig 3a); Nauru and Haiti had the highest and next highest ASMR and ASDR (Fig 3 b and c). It is noteworthy that the Marshall Islands and Federated States of Micronesia’s ASIR, ASMR, and ASDR are all among the top five of the 204 countries and territories (Fig 3).

**Fig 4:**
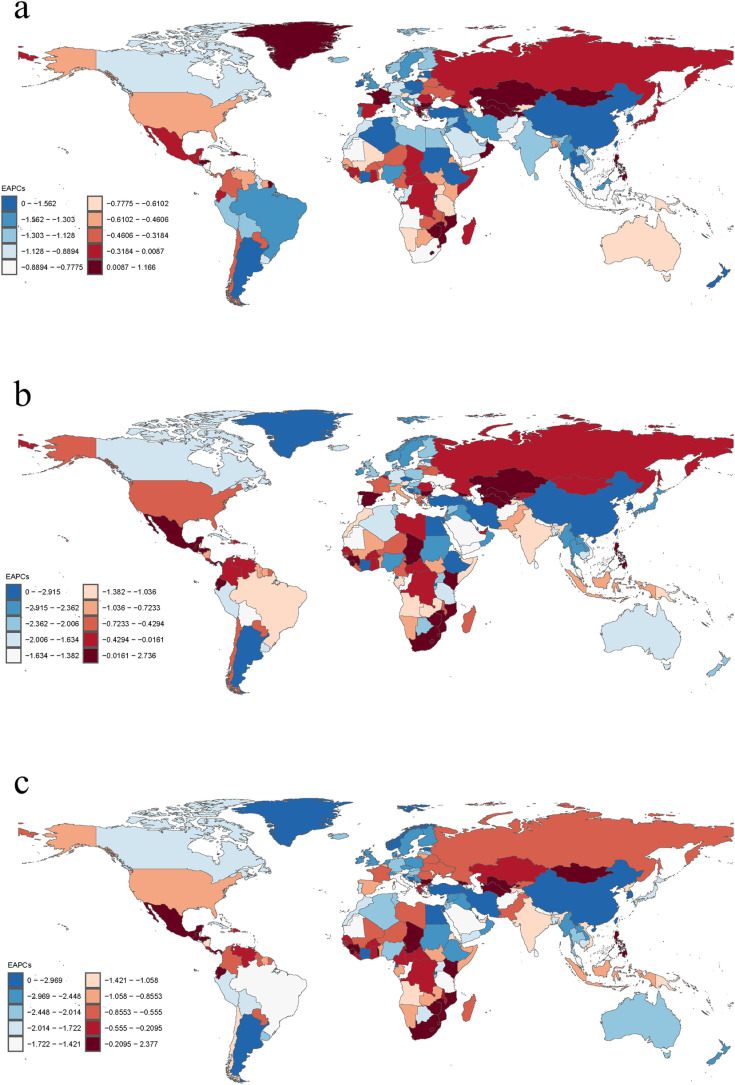
EAPC of age-standardized rates of subarachnoid hemorrhage by country, 1990-2021. (a) Age-standardized incidence rates; (b) age-standardized mortality rates; (c) age-standardized rates of DALYs.

During the period 1990–2021, ASIR, ASMR and ASDR declined in most of the 204 countries and territories in the world, with China showing the most significant downward trend, with the largest decline in ASIR, ASMR, and ASDR, and the Republic of Korea showing a significant decline, however, there are still about one-tenth countries and territories around the world, such as Turkmenistan, Zimbabwe, Georgia and Lesotho, also show an increasing trend (Fig 4).

### Attributing factor

**Fig 5:**
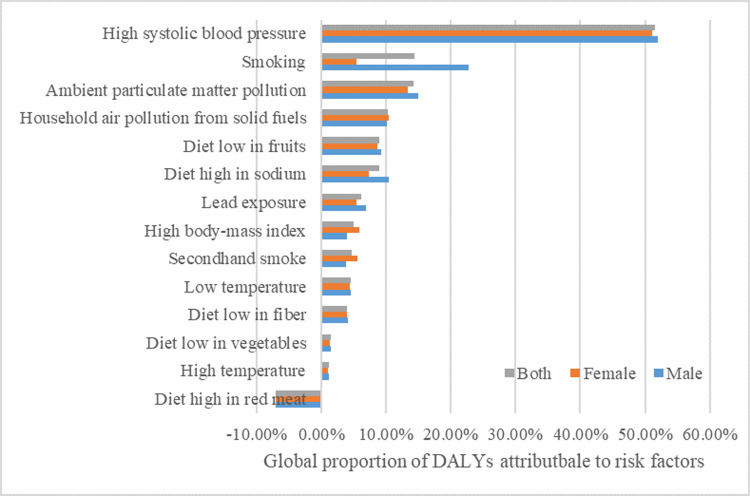
Global proportion of subarachnoid hemorrhage DALYs attributed to risk factors.

The proportion of global burden of disease attributable to risk factors for subarachnoid hemorrhage in 2021 is shown in the figure (Fig 5). The top five risk factors for subarachnoid hemorrhage globally for men and women of all ages are high systolic blood pressure (51.6% of total DALYs for subarachnoid hemorrhage), smoking (14.4% of total DALYs for subarachnoid hemorrhage), ambient particulate matter pollution (14.2%), pollution of household air from solid fuel (10.3%), and low fruit content in the diet (9.0%). For the male population, the top five risk factors changed: high systolic blood pressure (52.0% of the total DALYs for subarachnoid hemorrhage), smoking (22.7%), ambient particulate matter pollution (15.0%), a diet high in sodium (10.4%), and solid fuels for household air pollution (10.1%).For women, high systolic blood pressure (51.0% of total DALYs for subarachnoid hemorrhage), ambient particulate matter pollution (13.4%), household air pollution from solid fuels (10.4%), a diet low in fruits (8.7%), and a high sodium diet (7.4%) were the top five risk factors for subarachnoid hemorrhage. In contrast, a diet rich in red meat accounted for approximately -7.0% of total DALYs for subarachnoid hemorrhage globally for both sexes.

## Discussion

In our study, we estimated the global, regional, and national burden of subarachnoid hemorrhage (SAH), including its incidence, mortality, and DALY and its temporal trend, as well as DALYs attributable to 14 risk factors, using data from GBD 2021. Our analysis revealed that the global burden of subarachnoid hemorrhage was very heterogeneously distributed. From 1990 to 2021, the global incidence of subarachnoid hemorrhage increased by 37.09%, whereas the number of deaths decreased by 5.89% and DALYs counts decreased by 11.55%; age-standardized incidence, age-standardized mortality, and age-standardized DALY rates also demonstrated decreases, but the decrease in ASIR (EAPC = -1.52) was much less than that in ASMR (EAPC = -3.09) and ASDR (EAPC = -2.88), suggesting that prevention efforts for subarachnoid hemorrhage have been less substantial than treatment-related improvements, and that although subarachnoid hemorrhage accounts for fewer strokes than ischemic strokes and cerebral hemorrhages, we still need to strengthen prevention and control measures, given its high rate of disability. As the incidence of subarachnoid hemorrhage declines to a plateau in 2015–2021 due to an aging population, we need to adapt our prevention strategies to counteract this stagnation.

In the regional distribution of subarachnoid hemorrhage, we also observed a mismatch between incidence and mortality. In 2021 the high-income Asia-Pacific region had the highest age-standardized incidence rate (ASIR) for subarachnoid hemorrhage, but ASMR and ASDR were located in the middle of the range. Among the 21 global regions, Oceania and Andean Latin America have a higher burden of subarachnoid hemorrhage, with Oceanian countries such as the Marshall Islands, Solomon Islands, and the Federated States of Micronesia having the highest ASIR, ASMR, and ASDR of the 204 countries and territories. From 1990 to 2021, the burden of subarachnoid hemorrhage witnessed its most significant decline in East Asia, notably led by China, which demonstrated the highest percentage decrease among the 204 countries and territories, with the Republic of Korea also experiencing a substantial reduction. Nevertheless, approximately one-tenth of the world’s countries and territories, including Turkmenistan, Zimbabwe, Georgia, and Lesotho, continued to show increasing trends. While the burden of subarachnoid hemorrhage in sub-Saharan Africa remains relatively low, ASMR and ASDR have exhibited an upward trajectory over the past three decades. Consequently, the advancement of healthcare in low-income and lower-middle-income countries and regions will be paramount in addressing and mitigating the burden of this disease in the future [8].

Our study found that in 2021 SAH incidence will be highest among those aged 45–54 years and mortality will be highest among those aged 65–74 years. Combined with the results of a Bayesian model, the global incidence of SAH by 2041 may rebound due to population aging (from 9.3% of the population aged 65 years or older in 2021 to 16.2% in 2041) [9]. To meet the challenges of age differentiation and aging of the disease burden, a precise prevention and control system focusing on screening of high-risk groups and interventions throughout the life cycle needs to be constructed: secondary prevention such as screening for unruptured aneurysms should be promoted for the senior population, while primary prevention such as monitoring of hypertension, weight management, and low-sodium diets should be reinforced in the younger age groups.

In general, the incidence, mortality and DALY rates of subarachnoid hemorrhage in both men and women show an increase with age and are higher in men than in women at most ages, but there is no significant difference in the proportion of decline in ASIR, ASMR and ASDR between men and women over the last three decades. The incidence increases with age to a greater extent in menopausal women than in men due to diminished estrogen, higher incidence of atrial fibrillation (AF), and advanced age, and between the ages of 50 and 69 years, the incidence of subarachnoid hemorrhage are greater in women than in men and tends to have a poorer prognosis, consistent with past studies observing that the incidence of SAH increases more in women over the age of 55 years [10]. The latest gender-specific prognostic modeling showed that female SAH patients had a 1.3-fold increased risk of poor prognosis than men, which may be associated with vascular endothelial dysfunction due to the sudden drop in estrogen levels after menopause. The Zhejiang University team further found that the prognosis of female patients was more likely to be affected by intraventricular hemorrhage (OR=2.1) and delayed cerebral ischemia (OR=1.8), whereas the prognosis of males was mainly related to complications (e.g., lung infection). This discrepancy calls for the inclusion of gender-specific intervention pathways in clinical guidelines [11]. Risk factors for subarachnoid hemorrhage show differences between men and women, with the top five risk factors for the male population being: high systolic blood pressure, smoking, ambient particulate matter pollution, a high sodium diet, and solid fuel contamination of the home air, while for women, high systolic blood pressure, ambient particulate matter pollution, solid fuel contamination of the home air, a diet low in fruit, and a high sodium diet are the top five risk factors. The overall top five risk factors for subarachnoid hemorrhage are high systolic blood pressure (51.6% of total DALYs for subarachnoid hemorrhage), smoking, environmental particulate matter pollution, solid fuel pollution of home air, and diet low in fruit. It has also been suggested that secondhand smoke exposure is a risk factor for stroke [12,13]. Interestingly, the percentage of DALYs attributable to a diet rich in red meat was observed to be -7.03% (95% UI: -29.02% to 9.97%) in our study in the subarachnoid hemorrhage population. A meta-analysis in recent years confirmed [14]that consumption of processed red meat increased the incidence of hemorrhagic stroke (RR = 1.16), whereas the RR between consumption of fresh red meat and hemorrhagic stroke was 0.88, and another study in the same year [15]similarly demonstrated that there was no significant association between the consumption of unprocessed red meat and a reduced risk of hemorrhagic stroke; however, another dose-response meta-analysis showed that significant risk of stroke was observed when consumption of total red meat was higher than 50 g/day, processed red meat consumption was higher than 0 g/day and fresh red meat was more than 70 g/day [16].

In summary, by 2021, stroke maintains its position as the third leading cause of death globally and the fourth leading contributor to disability. Although the burden of subarachnoid hemorrhage has decreased over the past three decades, geographical disparities persist. To address this challenge effectively, we must take action on two fronts. First, in tackling the risk factors associated with subarachnoid hemorrhage, it’s imperative to tailor management and intervention strategies to their specific characteristics and evolving trends. Continuous efforts are needed to promote the widespread adoption of population-wide primary prevention strategies, aiming to reduce the overall incidence of SAH [17]. Secondly, to address the potential long-term health effects of subarachnoid hemorrhage, targeted and cost-effective treatment, care, and rehabilitation programs must be developed. Particular attention should be given to countries and regions with lower levels of economic development and limited healthcare resources. Our commitment lies in reducing the burden of disease and enhancing the quality of life for affected individuals, while advancing health equity on a global scale.

### Limitations of the study

Our study described and analyzed in detail the global, regional, national, and district burden of disease and risk factors for subarachnoid hemorrhage. There are some limitations to our study; most countries lack original, high-quality epidemiologic data on subarachnoid hemorrhage, this could lead to uncertainty in estimates and potential underestimation of the burden of SAH. In addition, our study was derived from the GBD 2021 database, thus potentially limiting the limitations of the GBD’s estimates of risk and risk factors [7], such as the lack of disaggregation of fresh or processed red meat in the assessment of diets rich in red meat, and the necessity for future iterations of the GBD to broaden the range of risk factors.
